# Psychometric Evaluation of 5- and 4-Item Versions of the LATCH Breastfeeding Assessment Tool during the Initial Postpartum Period among a Multiethnic Population

**DOI:** 10.1371/journal.pone.0154331

**Published:** 2016-05-02

**Authors:** Ying Lau, Tha Pyai Htun, Peng Im Lim, Sarah Ho-Lim, Piyanee Klainin-Yobas

**Affiliations:** 1 Department of the Alice Lee Centre for Nursing Studies, Yong Loo Lin School of Medicine, National University of Singapore, Singapore, Singapore; 2 Nursing Department, National University Hospital, Singapore, Singapore; 3 Department of Obstetrics and Gynaecology, National University Hospital, Singapore, Singapore; TNO, NETHERLANDS

## Abstract

**Objectives:**

The aim of this study was to evaluate the internal consistency, structural validity, sensitivity and specificity of the 5- and 4-item versions of the LATCH assessment tool among a multiethnic population in Singapore.

**Methods:**

The study was a secondary analysis of a subset of data (n = 907) from our previous breastfeeding survey from 2013 to 2014. The internal consistency of the LATCH was examined using Cronbach’s alpha. The structural validity was assessed using an exploratory factor analysis (EFA), and the proposed factors were confirmed by confirmatory factor analysis (CFA) using separate samples. Receiver operating characteristic analysis was used to evaluate the sensitivity and specificity of the LATCH score thresholds for predicting non-exclusive breastfeeding.

**Results:**

The Cronbach’s alpha values of the 5- and 4-item LATCH assessments were 0.70 and 0.74, respectively. The EFA demonstrated a one-factor structure for the 5- and 4-item LATCH assessments among a randomized split of 334 vaginally delivered women. Two CFA of the 4-item LATCH demonstrated better fit indices of the models compared to the two CFA of the 5-item LATCH among another randomized split of 335 vaginally delivered women and 238 cesarean delivered women. Using cutoffs of 5.5 and 3.5 were recommended when predicting non-exclusive breastfeeding for 5- and 4-item versions of the LATCH assessment among vaginally delivered women (n = 669), with satisfactory sensitivities (94% and 95%), low specificities (0% and 2%), low positive predictive values (25%) and negative predictive values (20% and 47%). A cutoff of 5.5 was recommended to predict non-exclusive breastfeeding for 5- and 4-item versions among cesarean delivered women (n = 238) with satisfactory sensitivities (93% and 98%), low specificities (4% and 9%), low positive predictive values (41%) and negative predictive values (65% and 75%). Therefore, the tool has good sensitivity but poor specificity, positive and negative predictive values.

**Conclusions:**

We found that the 4-item version demonstrated sound psychometric properties compared to the 5-item version. Health professionals can use the 4-item LATCH as a clinical tool because it is a concise, easy-to-use and valid tool for assessing breastfeeding techniques among a multiethnic population.

## Introduction

Breastfeeding is the best way to provide infants with the nutrients they need given the strong evidence of short- and long-term benefits [1, 2]. Although breastfeeding is the recommended method, successful breastfeeding can be a complex task for mother-infant dyads [3]. Currently, postnatal women stay in the hospital an average of 2–3 days, whereas previously postnatal mothers had longer lengths of hospital stay until breastfeeding was established [4]. Therefore, health care professionals have decreased opportunities to assess breastfeeding and to teach the proper techniques [4]. Hospitals have a great responsibility for the assessment of breastfeeding and for the determination of mother-infant dyads at risk for early weaning. Health care professionals should closely monitor pairs of mothers and infants to support their feeding techniques. Early assessment and appropriate management of breastfeeding is important to facilitate the success of breastfeeding mother-infant dyads.

Effective breastfeeding techniques can be divided into essential parameters that are important for overall success [5], but two review papers [6, 7] emphasized that no general consensus was found regarding the parameters comprising successful predictors. The commonly used parameters include rooting, latching, active sucking, audible swallowing and correct positioning [8, 9], all which were identified in a study of the objective predictors of successful breastfeeding [10]. To determine whether an infant is able to feed successfully, one must begin with an accurate assessment of feeding readiness and a thoughtful progression to full oral feeding [11]. Helping mothers to assess breastfeeding techniques was strongly related to breastfeeding success [5]. Thus, an accurate assessment tool is essential. The value of an assessment tool greatly depends on its sound psychometric properties [6]. The assessment tool must be short, sensitive and quick to assess breastfeeding techniques in clinical setting. An assessment tool that constitutes a checklist of essential parameters is needed [7, 11]. The use of a tool can facilitate important and appropriate assessment documentation for meaningful communications to the mother and other health care professionals [11].

LATCH was developed to identify five parameters for clinical assessment of breastfeeding techniques [12]. The letters of the acronym LATCH designate five separate assessment parameters: “L” for how well the infant latches onto the breast, “A” for the amount of audible swallowing, “T” for the mother’s nipple types, “C” for the mother’s level of comfort, “H” for the amount of support the mother has be given to hold her infant to the breast [12]. This tool was designed to be completed by the nursing staff and the mother’s self-report in response to standardized questions for each parameter. The score would thus reflect the degree of assistance needed from staff members for mother-infant dyads during breastfeeding so that health care professionals can assign priority for providing breastfeeding assistance. This assessment is a systematic documentation and standardized communication tool among health care professionals [12], and such a tool can assist in assessing breastfeeding technique using maternal and neonatal contributions to the breastfeeding process [13]. The LATCH is useful for health care professionals to determine priorities in providing maternal care and teaching [4].

However, the LATCH had inconsistent results for psychometric properties [6, 7]. Although the internal consistency [14], construct validity [15], sensitivity and specificity [16] for the LATCH were tested in previous studies, there were several limitations intrinsic to these studies regarding smaller sample size (n = 23) [15] among a specific population (women with low birth weight babies [14] or intrapartum complications [16]). LATCH had been used in modified versions (i.e., LAC and LACTHR) to investigate breastfeeding efficacy in a previous study, but this study did not report the psychometric properties of the modified versions [17]. The psychometric testing for the English version of the LATCH was conducted among English-speaking populations in the US [12, 15, 18], and the majority of samples were mostly from the Caucasian ethnic group, while a few reported other ethnic groups. Singapore is a multiethnic country in Southeast Asia [19], and 80% of the population is English-speaking [20]. Hence, this population represents a unique opportunity to explore the psychometric properties of the English version of the LATCH among multiethnic groups. To our knowledge, the structural validity of the LATCH assessment tool has not yet been investigated. To facilitate evidence-based assessment of breastfeeding techniques, further psychometric tests should be conducted among a larger sample with a multiethnic population to improve the strength of the external validity [6]. Therefore, the purpose of the present study was to evaluate the internal consistency, structural validity, sensitivity and specificity of the LATCH assessment among a multiethnic population in Singapore.

## Methods

### Design

This study is cross-sectional and quantitative in design. Data for the secondary analysis were obtained from the sample subset from our previous large breastfeeding survey [21] from 2013 to 2014 in Singapore. All relevant data were provided in (S1 File). We filtered data to remove any cases with preterm deliveries because there were significant differences in the sucking patterns between full-term and preterm babies [22] that might affect the effective breastfeeding technique [5]. Secondary analyses evaluated internal consistency, structural validity, sensitivity and specificity of the LATCH using separate samples in four stages.

### Setting

Participants in this study were recruited from a tertiary hospital with a delivery rate of 2935/year in Singapore. This regional public hospital provides comprehensive obstetric services to women and children of different demographic and socio-economic groups. Singapore is a multiethnic country in Southeast Asia with a population of more than 5.39 million people and covering an area of 716.1 km^2^ [23]. Chinese (74.3%) form the majority of the population, followed by Malays (13.3%), Indians (9.1%) and those from other ethnic groups (3.3%) [24]. Singapore is a multi-ethnic, multi-cultural cosmopolitan society interacting in a compact physical space and sharing many common dietary and lifestyle influences. Hence, Singapore represents a unique opportunity to explore the psychometric properties of the English version of the LATCH among multiethnic groups in Singapore.

### Participants and Sample

Convenience sampling was used in our previous large breastfeeding survey [21]. A sample of 907 mother-baby dyads was used in this secondary data analysis after preterm deliveries were excluded. The minimal sample size for exploratory factor analysis (EFA) was 200 [25], and the minimum necessary sample size for the confirmatory factor analysis (CFA) was 180 according to the proposed ratios of sample size to parameters estimates of 10 to 1 [26]. Therefore, we used separate samples for different psychometric evaluations, and each sample size was more than 200.

Evidence showed that the breastfeeding patterns of mothers were different according to the mode of delivery [27]; therefore, it was necessary to investigate breastfeeding techniques separately among vaginally and cesarean delivered women. EFA and CFA were conducted to test the structural validity of LATCH using separate samples [28]. Thus, the entire sample (N = 907) was categorized into vaginally delivered women (n = 669) and caesarean delivered women (n = 238). The vaginally delivered women (n = 669) were randomly split into two separate samples (n = 334 and n = 335).

The inclusion criteria for the participants included: (1) postpartum women who delivered babies in two postpartum wards, (2) ≥ 37 weeks of gestation and (3) English speaker. The exclusion criteria included: (1) maternal severe psychiatric illnesses, (2) women with major breast surgery (i.e., mastectomy or breast reduction) preventing establishment of effective breastfeeding and (3) infant transfer to the neonatal intensive care unit.

### Data Collection

Our previous large breastfeeding survey [21] was reviewed and approved by the hospital ethics approval board (Reference No: 2013/00513). Women who delivered babies during hospitalization in two postpartum wards within the data collection period of September 2013 to August 2014 were screened. Each eligible postpartum woman was invited to participate in the study. A full explanation of the study was given using each patient’s information sheet. Study participation was voluntary, and the respondent’s confidentiality was assured. The completed questionnaires were de-identified to prevent a respondent’s identity from being connected with the information. One experienced research assistant assessed the five parameters of the LATCH for participants within 48 to 72 hours after delivery.

### Instruments

LATCH [12] evaluated breastfeeding techniques based on observations and descriptions of effective breastfeeding. The tool assigned a numerical score of 0, 1 or 2 for these five parameters [12]. The ‘L’ assessment was scored as “2” if good latching is identified (grasps breast, tongue down, lips flanged and rhythmic sucking); “1” if repeated attempts to hold the nipple in the mouth or to stimulate to suck were identified; and “0” if poor latching (too sleepy or reluctant or no latching achieved). The ‘A’ assessment was scored as “2” if audible swallowing occurred (spontaneous and intermittent <24 hours old or spontaneous and frequent >24 hours old), “1” if a few swallows occurred with stimulation and “0” if ineffective swallowing occurred. The ‘T’ assessment is scored as “2” if an everted nipple was present (after stimulation), “1” if the nipple was flat and ‘0’ if the nipple was inverted. The ‘C’ assessment was scored as “2” if the breast was soft and tender, “1” the breast was filled or reddened / featured small blisters / bruised nipples and “0” if the breast was engorged or if a cracked appeared. The ‘H’ assessment was scored as “2” if good positioning was achieved (no assistance from the staff or mother able to position / hold infant), “1” if minimal assistance was required (i.e., elevate the head of the bed or place pillows for support) and “0” if full assistance was required (staff holds the infant at the mother’s breast) [12]. The total score ranged from 0 to 10, with the higher score representing efficient breastfeeding techniques [12]. Two feeding categories were used: “exclusive breastfeeding” (only breast milk, including expressed milk) and “non-exclusive breastfeeding” (the sum of the partial and artificial feeding). Mother-infant characteristics (age, race, parity and sex of babies) were collected from the medical records.

### Data Analysis

IBM SPSS Statistics 22.0 (IBM Corporation, Armonk, NY, USA) was used for the data analysis. Analyses were conducted in four stages. The analytic strategies as used in this study are described in detail below.

### Exploratory Factor Analysis

In the first stage, a EFA was conducted to test structural validity [29] using the principal axis factoring (PAF) extraction method among a randomized split of 334 vaginally delivered postpartum women. In PAF, the analysis of data structure focused on shared variance that was unique to individual measurements [30, 31]. A factor loading (λ) > 0.3 was considered for each variable onto each factor [32]. Bartlett’s Test of Sphericity assessed the hypothesis that the correlation matrix was an identity matrix for the factor analysis, and p <0.05 indicated suitable significance for structure detection [33]. The Kaiser-Meyer-Olkin (KMO) measure of Sampling Adequacy tested the proportion of variance in our variables. A KMO of 0.6 was suggested as the minimum value for good factor analysis [34].

### Internal Consistency

The reliability of the LATCH assessment was first tested among a randomized split of vaginally delivered women (n = 334) using Cronbach’s alpha reliability with correlation coefficients ≥ 0.7 [35] and corrected item-to-total correlation coefficients > 0.3 [36] taken as the criterion value. Cronbach’s alpha is an index of reliability to test for internal consistency, which refers to the degree of homogeneity of items that measure the same dimension [37].

### Confirmatory Factor Analysis

To further confirm the construct of the LATCH, a CFA using the Analysis of Moment Structures (AMOS) software (version 22.0) was performed [38] among another set randomized split of vaginally delivered postpartum women (n = 335) and cesarean delivered women (n = 238) to confirm the proposed model based on a priori information of the EFA in different modes of delivery. Model goodness-of-fit indices were used to evaluate the model fit: the Goodness-of-fit Index (GFI), the Adjusted Goodness-of-fit Index (AGFI), the Incremental Fit Index (IFI), the Tucker-Lewis Index (TLI), the Comparative Fit Index (CFI) and the Root Means Square Error of Approximation (RMSEA) [38, 39]. Cut-off criteria for the fit indexes were used: (1) GFI > 0.9, (2) AGFI > 0.9, (3) IFI > 0.9, (4) TLI > 0.9, (5) CFI > 0.9, and (6) RMSEA < 0.08 [40–42].

### Sensitivity, Specificity and Predictive Values

A receiver operating characteristic analysis was used to evaluate sensitivity (true positive rate) and specificity (true negative rate) of the LATCH score thresholds for predicting non-exclusive breastfeeding [43]. Because mode of delivery [27] is a significant factor of the breastfeeding pattern, separate data analyses were used among vaginally (n = 669) and caesarean (n = 238) delivered women. Sensitivity referred to the capacity of the LATCH assessment tool to correctly identify postnatal women who would be at risk of non-exclusive breastfeeding, whereas specificity referred to the capacity of the LATCH assessment tool to correctly identify postnatal women who would not be at risk of non-exclusive breastfeeding [43]. Positive predictor values (PPV) referred to the proportion that postnatal women actually were non-exclusive breastfed using LATCH assessment tool, whereas negative predictor values (PPV) referred to the proportion that postnatal women actually were exclusive breastfed using LATCH assessment tool [43]. Likelihood ratios (LRs) referred to ratio between true positive rate (sensitivity) and false positive rate (1 –specificity) [43]. There was a trade-off between the sensitivity and specificity of a clinical tool [43]. A perfect predictor would be described as 100% sensitive and 100% specific [44]. The Youden’s index [*J* = Sensitivity + Specificity—1] was calculated as a reference for the suitability of the cutoff point [45]. The highest index *J* was selected as the “best” cutoff point for our data set [45].

## Results

A total of 907 women-infant dyads were used in this secondary data analysis. The mean age of the women was 30.83 years (SD = 4.53), and the ethnic composition of women was Chinese = 40.0%, Malays = 22.6%, Indians = 22.7% and others = 14.7%. More of than half were multiparous (51.6%). The mean with standard deviation of infant body weight was 3.14±0.39 kilograms, and 52.6% of the infants were male. The proportions of exclusive breastfeeding and non-exclusive breastfeeding were 70.5% and 29.5% among the entire sample (n = 907), 74.3% and 25.7% among vaginally delivered women (n = 669), and 59.7% and 40.3% among caesarean delivered women (n = 238), respectively.

### Exploratory Factor Analysis

Table 1 investigates the factor loading of EFA; however, component “C” had factor loadings (λ = 0.28) less than the criterion value of 0.3 [32], suggesting low communalities. After examining the meaning of this component in terms of specificity, it was determined that the item representing the mother’s degree of breast or nipple comfort might not consider only the breastfeeding techniques specifically. We subsequently extracted this component from the original 5-item version to form a 4-item version to compare their internal consistency, factor loadings, sensitivity and specificity as shown in Tables 1–3.

**Table 1:** Exploratory factor analysis of the 5- and 4-item LATCH breastfeeding assessment tools using principal axis factoring extraction method among vaginally delivered women (n = 334).

	LATCH	5-item	4-item
L:	Latch	0.77	0.79
A:	Audible swallowing	0.65	0.65
T:	Type of nipple	0.42	0.42
C:	Comfort	0.28	-
H:	Hold	0.76	0.75
Eigenvalues		1.85	1.78
Total variance explained		37.07%	44.45%
Kaiser-Olkin Measure of Sampling Adequacy		0.76	0.75
Bartlett’s Test of Sphericity:		p < 0.0001	p < 0.0001

**Table 2:** Descriptive statistics and reliability tests of the 5- and 4-item LATCH breastfeeding assessment tools among vaginally delivered women (n = 334).

LATCH		5-item				4-item		
		M (SD)	Corrected	α	α	Corrected	α	α
			Item-to-Total Correlation	(if item deleted)		Item-to-Total Correlation	(if item deleted)	
L:	Latch	1.85 (0.38)	0.61	0.60		0.64	0.62	
A:	Audible swallowing	1.86 (0.37)	0.53	0.63		0.53	0.67	
T:	Type of nipple	1.80 (0.45)	0.36	0.70	0.70	0.38	0.75	0.73
C:	Comfort	1.83 (0.40)	0.24	0.73		-	-	
H:	Hold	1.69 (0.54)	0.61	0.58		0.61	0.63	

M (SD) = Mean (standard deviation); α = Cronbach’s Alpha

**Table 3:** Cutoff values of the 5- and 4-item LATCH breastfeeding assessment tools in detecting non-exclusive breastfeeding for vaginal (n = 669) and caesarean (n = 238) deliveries.

	LATCH cutoff score	Specificity (true negative rate)	Sensitivity (true positive rate)	*J*	Positive predictive value (PPV)	Negative predictive value (NPV)	Likelihood ratio (LRs)
Vaginally delivered women (n = 669)							
	**5.5**	**2%**	**94%**	**-0.04**	**25%**	**47%**	**0.96**
5-item	6.5	4%	84%	-0.12	23%	40%	0.87
Range:	7.5	9%	78%	-0.14	23%	53%	0.85
0–10	8.5	21%	67%	-0.12	23%	64%	0.84
	9.5	43%	47%	-0.11	22%	70%	0.81
	**3.5**	**0%**	**95%**	**-0.04**	**25%**	**20%**	**0.96**
4-item	4.5	3%	90%	-0.07	24%	48%	0.93
Range:	5.5	7%	80%	-0.14	23%	48%	0.85
0–8	6.5	16%	72%	-0.13	23%	61%	0.85
	7.5	38%	52%	-0.10	22%	69%	0.83
Cesarean delivered women (n = 238)							
	**5.5**	**4%**	**98%**	**0.02**	**41%**	**75%**	**1.02**
5-item	6.5	8%	94%	0.01	41%	65%	1.02
Range:	7.5	15%	87%	0.01	41%	62%	1.02
0–10	8.5	26%	70%	-0.04	39%	56%	0.94
	9.5	44%	50%	-0.06	38%	57%	0.90
	3.50	1%	98%	-0.01	40%	50%	0.99
4-item	4.50	4%	96%	0.00	40%	60%	1.00
Range:	**5.50**	**9%**	**93%**	**0.02**	**41%**	**65%**	**1.02**
0–8	6.50	18%	76%	-0.06	39%	53%	0.93
	7.50	38%	56%	-0.06	38%	56%	0.91

*J* = Youden's index [sensitivity + specificity-1]; Values for optimal cutoff points are given in bold.

### Internal Consistency

We tested internal consistency and corrected item-to-total correlations among the first set of vaginally delivered women (n = 334). The descriptive analysis and internal consistency of the LATCH assessment tool are shown in Table 2. The corrected item-to-total correlation of the 5-item ranged from 0.24 to 0.61. The corrected item-to-total correlation of component “C” was 0.24, which was less than the criterion value of 0.3 [36]. If we deleted this component, the alpha would improve to 0.73, and the overall Cronbach’s α would improve from 0.70 to 0.74, values that are close to the criterion value of 0.7 [35], suggesting acceptable internal consistency [35].

The EFA suggested a one-factor model using the PAF extraction method. The factor loadings of each item are shown in Table 1. The factor loading ranged from 0.28 to 0.77 for the 5-item version and 0.42 to 0.79 for the 4-item version. The KMO measures for the data were 0.76 and 0.75, respectively, which suggested that these data were suitable for factor analyses, as the measures exceeded the recommended value of 0.60 [34]. The factorability of the correlation matrix was supported by Bartlett’s test of sphericity for research statistical significance (p < 0.001) [33]. The 4-item version showed a total variance higher (37.07%) than the 5-item version (44.45%).

### Confirmatory Factor Analysis

**Fig 1:**
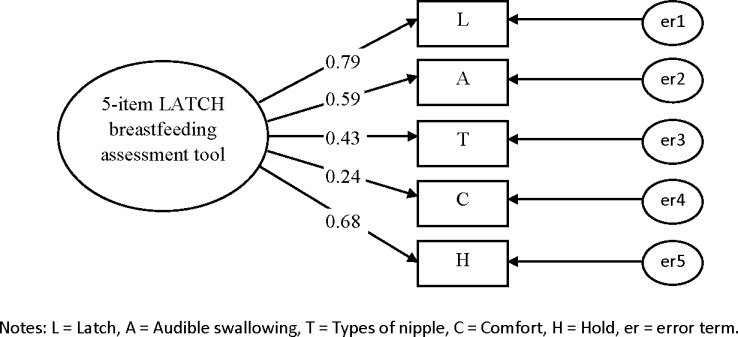
Confirmatory factor analysis for the 5-item LATCH breastfeeding assessment tool among vaginally delivered women (n = 335). Fit indices: GFI=0.980; AGFI=0.941; IFI=0.957; TLI=0.913; CFI=0.956; RMSEA=0.086.

**Fig 2:**
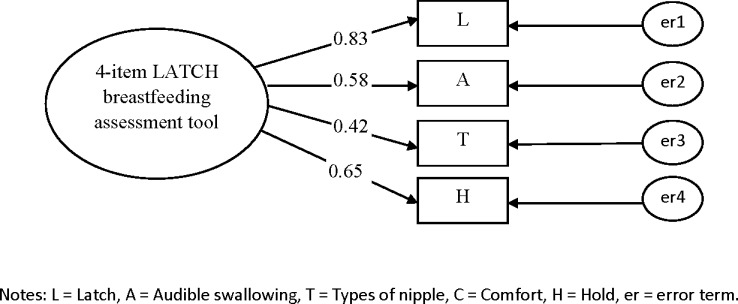
Confirmatory factor analysis for the 4-item LATCH breastfeeding assessment tool vaginally delivered women (n = 335). Fit indices: GFI=0.995; AGFI=0.976; IFI=0.995; TLI=0.985; CFI=0.995; RMSEA=0.044.

**Fig 3:**
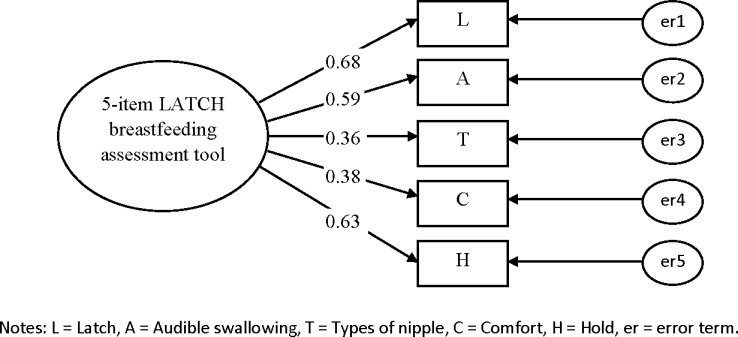
Confirmatory factor analysis for the 5-item LATCH breastfeeding assessment tool among caesarean delivered women (n = 238). Fit indices: GFI=0.961; AGFI=0.883; IFI=0.889; TLI=0.772; CFI=0.886; RMSEA=0.125.

**Fig 4:**
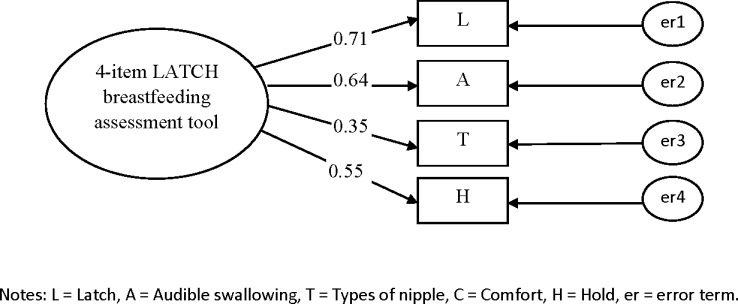
Confirmatory factor analysis for the 4-item LATCH breastfeeding assessment tool among caesarean delivered women (n = 238). Fit indices: GFI=0.994; AGFI=0.971; IFI=0.993; TLI=0.979; CFI=0.993; RMSEA=0.042.

CFAs were conducted to determine whether the data were consistent with the specified model that had been suggested by the EFA among a second set of vaginally delivered women (n = 355) and caesarean delivered women (n = 238) to confirm whether the data fitted the model adequately for the 5- and 4-item LATCH assessment tools (Figs 1–4). Two CFAs of the 4-item LATCH breastfeeding assessment demonstrated good fit indices [40–42] to the data ([1] GFI = 0.994–0.995, [2] AGFI = 0.971–0.976, [3] IFI = 0.993–0.995, [4] TLI = 0.979–0.985, [5] CFI = 0.993–0.995, and [6] RMSEA = 0.042–0.044) compared to two CFAs of the 5-item LATCH breastfeeding assessment to the data ([1] GFI = 0.961–0.980, [2] AGFI = 0.883–0.941, [3] IFI = 0.889–0.957, [4] TLI = 0.772–0.957, [5] CFI = 0.886–0.956, and [6] RMSEA = 0.086–0.125).

The factor loadings (λ) of the 5-item LATCH assessment tool were 0.24–0.79 and 0.38–0.68 for vaginally (Fig 1) and cesarean (Fig 2) delivered women, and the factor loadings of the 4-item LATCH assessment tool were 0.42–0.83 and 0.35–0.71 for vaginally (Fig 3) and cesarean (Fig 4) delivered women, respectively. The parameter “C” (comfort level; λ = 0.24) had factor loadings less than the criterion value of 0.3 [32] among vaginally delivered women (n = 355), indicating that this parameter was not specific for overall breastfeeding techniques among vaginally delivered women.

### Sensitivity, Specificity and Predictive Values

Table 3 illustrates the dilemma created by the trade-off between sensitivity and specificity of the 5- and 4-item LATCH assessment tools to predict non-exclusive breastfeeding among vaginally (n = 669) and cesarean (n = 238) delivered women. Cutoff points of 5.5 and 3.5 were recommended for the 5- and 4-item tools among vaginally delivered women because of the highest index values of *J* (-0.04), with sensitivities of 94%– 95%, specificities of 0–2%, PPV of 25%, NPV of 20%– 47% and LRs of 0.96, as shown in Table 3.

A cutoff point of 5.5 was recommended for the 5- and 4-item tools among caesarean delivered women because of the highest index values of *J* (0.02), with sensitivities of 93%– 98%, specificities of 4%– 9%, PPV of 41%, NPV of 65%– 75% and LRs of 1.02. These results interpreted that the capacities of the 4- and 5-item LATCH assessment tools to identify 94% to 98% of all women who would be at risk of non-exclusive breastfeeding, but the tools were only enable to identify 0 to 9% of all women who would not be at risk of non-exclusive breastfeeding. Only 25% and 41% of postnatal women were actually true positive (non-exclusive breastfed), whereas 20% and 75% of postnatal women were actually true negative (exclusive breastfed) using a cutoff point of 3.5 and 5.5 for the 4- and 5-item tools. Therefore, the sensitivities of the 5- and 4-item LATCH assessment tools were satisfactory but had poor specificity, PPV and NPV.

## Discussion

To our knowledge, the present study describes the first multiethnic sample subjected to LATCH assessment and was the first to perform internal consistency, structural validity, sensitivity and specificity evaluations of the 5- and 4-item LATCH tools in this population.

### Internal Consistency

The Cronbach’s alpha values of the 5- and 4-item LATCH assessment tools ranged from 0.70 to 0.74, indicating acceptable internal consistency in this study. These results were inconsistent with a previous study [14], where the Cronbach’s alpha of the LATCH assessment was 0.93 among 85 women who had low birth weight babies in Turkey [14]. The difference in the internal consistency might be related to the different perceptions used to measure the parameters of breastfeeding techniques among nursing staff across cultures. The discrepancy might also be due to the differences in nature between the general subjects (women with full-term babies) in our study and the specific subjects (women with low birth weight babies) in the previous study [14] in Turkey, which might have influenced the responses to the items on the scale. Another possibility for why the present study reported a lower internal consistency that might be related to the small number of items included in the LATCH assessment [46]. Evidence has shown that the higher the correlations are among more items on a scale, the higher the value of Cronbach’s alpha will be [47]; therefore, Cronbach’s alpha could be affected by the length of the scale [46]. Therefore, it was unsurprising that the 4- and 5-item LATCH assessment tools had lower Cronbach’s alpha values in our study.

### Structural Validity

To the best of our knowledge, the present study is the first report to provide evidence of the structural validity of the original version (5-item) and the simplified version (4-item) of the LATCH assessment tool. The structural validity revealed a one-factor structure for the 4- and 5-item LATCH assessment tools by EFA and CFA in separate samples of this study. The CFA confirmed that the construct of the 4-item version yielded better model fit indices compared to the 5-item version. The parameters, “L”, “A”, “T”, and “H”, with relevant contents, appeared to cluster well regarding breastfeeding techniques compared with the 4-item LATCH, with satisfactory factor loadings. This finding might be due to the parameters of good latching, effective swallowing, the shape, size and texture of the nipple and the correct position of the infant’s on the breast, as these factors are critical in establishing successful breastfeeding exclusivity [8, 9].

However, parameter “C” had unsatisfactory factor loadings among vaginally delivered women [32]. One possible reason for this result was the different rating standard for component “C” [15]. The assessment of parameter “C” included both breast and nipple areas [12]. The raters thought that a filling breast during the first week postpartum would be a positive sign that things were going well, so they tended to rate such an observation as “2” instead of “1” [15]. Another possible interpretation of this result was that the comfort level might not specifically reference breastfeeding techniques; breast comfort could also be related to breast support [48] or the mother’s subjective sensation [7]. Thus, the parameter “C” might not fit into the substantive content or meaning of breastfeeding techniques. Therefore, our study eliminated this parameter to form the 4-item version instead of the 5-item version. Nonetheless, this study is the first to explore the internal structure of the LATCH, and future investigation is necessary for verify its structural validity.

### Sensitivity, Specificity and Predictive Values

Receiver operating characteristic analysis confirmed the high sensitivities (few false negatives) of the 4- and 5-item versions of the LATCH assessment tool and indicated the proposed thread scores using the Youden’s index [43], but very low specificities (many false positives), PPV (few true positives) and NPV (few true negatives) were obtained. Cutoffs of 5.5 and 3.5 were used to identify correctly postnatal women who would be at risk of non-exclusive breastfeeding for the 5- and 4-item versions among vaginally delivered women. A cutoff of 5.5 was used to correctly identify postnatal women who would be at risk of non-exclusive breastfeeding for the 5- and 4-item versions among cesarean delivered women. This finding was lower than the cutoff values of 9 to 10 among high-risk women, women who had cesarean delivery, vaginal delivery with primiparity and phototherapy in a previous study [16]. However, it was impossible to perform a comparison, as the nature of the two populations was different. Although the specificities of the two versions of the LATCH were not satisfactory, a high sensitivity was clearly important where the LATCH assessment tool was used to correctly detect these high-risk groups during short-term postnatal hospitalization [44] because these non-exclusive breastfed women were in need of professional assistance [16, 49].

In sum, the 4-item LATCH provides clinically meaningful parameters that may be useful for a more accurate assessment of the dimensions of the breastfeeding techniques among a multi-ethnic population. Therefore, the 4-item LATCH is recommended as an alternative choice for a clinical tool because it is brief, valid, reliable and sensitive.

### Implications

A scientifically sound breastfeeding clinical tool is critical for evaluating breastfeeding techniques to enable both clinicians and researchers to identify, monitor, and manage feeding problems occurring in the initial postpartum period [6]. The use of the LATCH assessment tool as an objective means of assessing breastfeeding can assist nurses in recognizing the critical maternal and infant variables essential in the early breastfeeding process, defining areas of need, and determining priorities in assisting and teaching. Short hospital stays are challenging to assess breastfeeding and to identify those who are at risk for breastfeeding problems due to limited time [4]. Perhaps it is time to examine how the post-discharge breastfeeding supports have functioned in this respect. Continuing the application of the LATCH assessment tool in the community after discharge may provide a consistent approach that may improve the care for the breastfeeding mother-infant dyad. Further innovative strategies are warranted to customize hospital programs promoting breastfeeding counseling and to tailor them to successful breastfeeding by mothers during and after discharge from the hospital.

### Limitations

One limitation of this study was its cross-sectional design using a convenient sampling method in a single hospital-based setting, which limited the generalization of findings. Scoring the LATCH assessment tool within 72 hours of postpartum was also a limitation, as that time period is not considered sensitive enough to detect differences between groups within a longer postpartum period. The very low specificity, PPV and NPV of the tool might increase the high probability of false positives, low probability of true positive and true negative among non-exclusive breastfeeding women. Finally, this tool addressed only four to five parameters of breastfeeding techniques; infant test-weights and elimination patterns should be considered as additional parameters in further studies.

## Conclusions

The 4-item version of the LATCH is a short, sensitive, reliable and valid version that can be considered a routine assessment tool to assist mother-infant dyads in essential parameters of breastfeeding techniques. It would be most helpful to be able to identify those women at greatest risk for early weaning in hospital- and community-based settings so that they could receive tailored-made interventions and high-quality lactation support. Such efforts might ultimately enable effective breastfeeding techniques to achieve successful breastfeeding outcomes.

## Supporting Information

S1 File Data set for PLOS one (n = 907). (SAV)
